# Unlocking potential: Evaluating Nepal's cooperative-backed vegetable value chain

**DOI:** 10.1016/j.heliyon.2024.e40120

**Published:** 2024-11-07

**Authors:** Ghanashyam Khanal, Ratnesh Kumar Dev, Tek Maraseni, Niranjan Devkota, Udaya Raj Paudel

**Affiliations:** a Tribhuvan University, Nepal; b Quest International College, Pokhara University, Gwarko, Lalitpur, Nepal; c Zikipoint Health Private Limited, Sanepa, Lalitpur, Nepal; d Department of Economics, Patan Multiple Campus, Tribhuvan University, Patandhoka, Lalitpur, Nepal

**Keywords:** Value chain analysis, Cooperatives, Farmer, Traders, Value addition, Logistic model, Nepal

## Abstract

Cooperatives play a vital role in rural and agricultural development within value chains. They employ 279 million worldwide, and the top 300 earned $2.1 trillion in 2019, ranking as the 10th largest economy if considered a country. The study examines the current state of the vegetable value chain, the involvement of cooperatives, factors influencing value addition, and the challenges cooperatives encounter within the vegetable value chain. The research utilized structured questionnaires to gather data from 183 participants, comprising 164 farmers, 10 traders, and 9 cooperative members in Nepal's Siraha and Saptari districts. The collected data underwent analysis using a binary logistic regression model. The study found that there is no proper vertical and horizontal linkage among the chain actors. Cooperatives are good at facilitating access to credit, input, and training but their involvement in both input supply and credit services is limited. Among the 31 variables included in the logistic regression, 14 were deemed statistically significant. Particularly, the age of farmers, their off-farm income, access to information, and access to inputs could be instrumental to value addition in the VVC. The study suggests that early-stage co-innovative and collaborative value chains, with better input supply, business planning, and cooperative autonomy, can boost VVC profitability and sustainability. Partnerships and a standard code of conduct among chain actors and cooperatives can further enhance trust and innovation.

## 1 Introduction

Cooperatives are voluntary initiatives that pursue shared interests [1,2] and encourage rural and agricultural development [3]. They act as a linking device, reducing reliance on middlemen, promoting equal profit distribution, and fostering innovation [4]. Cooperatives engage in economic activities such as credit disbursement and input distribution [5]. The benefits of collective activity are shared among producers in patron-driven organizations [6,7], making cooperatives essential for value chain development and improving farmers' lives. According to the International Cooperative Alliance [8], cooperatives employ approximately 279 million people worldwide, accounting for around 10 % of the employed population. Moreover, the largest 300 cooperatives generated combined revenue of over USD 2.1 trillion in 2019, positioning them as the 10th largest economy if considered a country.

The value-chain network encompasses actions from product conception to the final consumer, including design, sourcing, marketing, and distribution [[9], [10], [11]]. It involves all operations in manufacturing, supplies, storage, processing, and distribution of commodities [4]. Smallholder self-organization promotes risk-sharing and value creation through coordination and integration [7]. Cooperatives contribute to value addition, market stability, and expansion by processing agricultural products and attracting new customers [12]. Cooperatives act as intermediaries with greater bargaining power, improving market access for farmers [13].

In the agricultural sector, cooperatives handle around 40 % of global agricultural production, playing a crucial role in ensuring food security and supporting rural development [8]. Cooperatives also contribute to financial inclusion by providing essential financial services to individuals who would otherwise be excluded from the formal banking system [14]. Additionally, cooperatives prioritize gender equality, with around 48 % of cooperative members worldwide being women, enabling them to participate in decision-making processes and achieve economic empowerment. Through their commitment to sustainability, cooperatives implement eco-friendly practices, support organic farming, and promote renewable energy initiatives [8]. These global figures and facts highlight the instrumental role of cooperatives in employment, economic growth, agriculture, financial inclusion, gender equality, and sustainable development.

Economically, Nepal is an agrarian country where most of the people (65.2 %) depend on agriculture for their livelihood [15]. As Chaudhary et al. [16] among the total population, 69.4 % has below 1 ha of landholders, indicating that Nepali farmers fall in the category of small and marginal farmers. Still, a quarter of the population (i.e., 25.2 %) is below the poverty line. Food production is not keeping pace with the increase in population. In this context, the type and capacity of organizations working in the agriculture sector have a big impact on the success of agricultural development projects [[17], [18], [19]]. The cooperative system looks to be the most effective alternative technique for involving people in development [20].

In Nepal, there is a growing emphasis on developing efficient agricultural value chains, leading to the emergence of creative and effective value chains [21]. The agro-food systems in Nepal are rapidly changing, and one prominent market trend is the establishment of integrated food supply chains. Farmers, processors, merchants, and other stakeholders in the value chain are collaborating to replace traditional food production methods with approaches like manufacturing processes [17,22]. This transformation incorporates productive changes and value addition at each stage of the value chain. Studies have shown that cooperatives play a vital role in supporting value chain development by facilitating market access, improving product quality, increasing bargaining power, strengthening connections between farmers and other value chain participants, and enhancing resilience to external shocks.

While there have been some studies on value chain processes in Nepal such as Niroja et al. [12], Chaudhary et al. [16], Sharma [19], Kalauni and Joshi [23], Baral et al. [24]; none of them precisely explore the case of vegetable value chain in the rural area of Nepal. To fulfill this gap, we analyze the status of the vegetable value chain, explore the role of cooperatives within it, investigate factors affecting the value addition (processes and actions that increase the economic value of vegetables) in the chain, and identify challenges faced by cooperatives in the vegetable value chain in the rural area of Siraha and Saptari districts of Nepal. The vegetable value chain includes input suppliers, producers, collectors/assemblers, brokers/middlemen, wholesalers, retailers, and consumers. Supporting actors like government agencies, NGOs, and cooperatives provide inputs, credit, training, and other support. Challenges faced by value chain actors include low market prices, post-harvest losses, and limited access to credit and market information. The study offers insights to inform policies and interventions for a more efficient, inclusive, and sustainable value chain system. For sure, by understanding the various actors and factors that shape the vegetable value chain in this context, stakeholders can work together to address the challenges faced by small-scale farmers and other actors and promote more equitable and sustainable value chain development in the region.

### 1.1 Value chain in agricultural products

In the food and agriculture industry, a value chain is defined as "a series of sequential and parallel operations or functions about the distribution, production, and selling of foodstuffs" [25]. The value chain examines the transit of food from farmers to distributors and retailers, considering the links that exist between participating actors (e.g., producers, suppliers, distributors, and consumers) [26]. Table 1 lists some references in the value addition process in different parts of the world.

**Table 1:** Value chain in agriculture product.

Study	Findings	
Nguyen and Thien [27]	Find that white-leg shrimp farming in Vietnam has a positive net return to farmers.	
Junior et al. [28]	Analyze the impact of value chain strategies on honey production growth in Brazil, finding that certain strategies contribute the most to performance.	
Wang [29]	Research on sustainable livelihoods in China reveals that the price premium of organic products is distributed to actors along the chain and that farmers experience significant financial capital improvement in organic farming.	
Amosi [30]	Study on the value chain of baobab products in Malawi concludes that actors in the baobab trade should increase market power, market share, and economies of scale through horizontal integration	
Kalauni and Joshi [23]	Research on large cardamom cultivation in Nepal recommends improving the production process and identifying potential international markets to help farmers receive higher prices.	

### 1.2 Value chain in vegetables

Vegetables, being one of the most valued horticultural commodities, can increase in price through value addition. Due to their perishable nature, they require special management in the supply chain and are classified as rapidly moving products, leading to lower inventory costs. Value chain analysis examines how value chains evolve in structure, conduct, and performance in response to market conditions, technologies, and regulations. Table 2 lists key literature on the vegetable value chain, detailing authors, study areas, methods, variables, results, and recommendations.

**Table 2:** Value chain in vegetables.

Author/Country	Methods and Data	Variables	Results	Conclusion
Srimanee and Routray [31]/Thailand	Secondary (Longitudinal method) and Primary data	*Dependent Variable*: Value chain *Independent Variables:* Policy, Supply chain management, Retailing	Supermarkets connect farmers to markets and improve cultivation practices to enhance product quality	The government has promoted both domestic and export markets to improve the FFV market and enhance production efficiency.
Blandon et al. [32]/Honduras	Qualitative method	*Dependent variable:* SSC for FFV, *Independent Variables:* human capital variables, farm characteristics and assets and transaction costs	Small-scale farmers can participate in new supply chains when certain conditions are met, including incentives, trust-based relationships, risk reduction practices, and collective action.	Farmers' organizations must invest collectively and follow joint planting and harvesting schedules.
Arah et al. [20]/Ghana	Qualitative method	*Dependent Variable:* Post-harvest Handling, *Independent Variables:* Harvesting, pre-cooling, grading, packaging, storing, and transportation	Harvesting, pre-cooling, grading, packaging, storing and transportation practices are vital for maintaining quality and extending shelf life.	Proper postharvest handling practices and treatments maintain fruit quality and extend shelf life while neglecting these practices leads to significant losses.
Mazhar et al. [33]/Pakistan	Quantitative and Qualitative analysis	*Dependent Variable:* Sustainable Livelihood, *Independent Variables:* Improving Vegetable Value Chains	The findings highlighted the need for improvement in the inconsistent quantity and quality of vegetables supplied to the market.	Partnerships with public and private institutions throughout the testing and application of value chain interventions are considered vital to sustain impacts.
Amaya et al. [34]/Guatemala	Qualitative, Rapid Market Appraisal (RMA) method		Low demand in the value chain is primarily hindered by factors such as limited consumer awareness, changing eating habits, restricted recipes, and availability in home-gardens.	Low demand and profitability discourage producers from commercially cultivating chaya, and vendors are reluctant to sell it as a result.

Table 2 summarizes studies on the vegetable value chain across various countries. Srimanee and Routray [31] in Thailand found that government policies and supermarkets enhance market connections and cultivation practices. Blandon et al. [32] in Honduras emphasized the role of incentives, trust, and collective action for small-scale farmers' participation in supply chains. Arah et al. [20] in Ghana highlighted the importance of post-harvest handling for quality maintenance and shelf life extension. Mazhar et al. [33] in Pakistan identified the need for partnerships to improve vegetable quality and supply consistency. Amaya et al. [34] in Guatemala noted that low consumer demand and profitability hinder the commercial cultivation of certain vegetables.

## 2 Methodology

### 2.1 Conceptual framework

This study intertwines various theories, including Value Chain Theory, Social Network Theory, Game Theory, Stakeholder Theory, Cooperative Theory, and Organizational Change Theory, to understand the role of cooperatives and value chains [11,[35], [36], [37], [38]].

***Value Chain Theory****:* This theory is central to understanding how cooperatives influence each stage of the vegetable value chain, from input supply to consumer. It provides a framework for analyzing how cooperatives enhance value creation, reduce inefficiencies, and improve market access for smallholder farmers. By examining the entire process, this theory helps in identifying the roles and impacts of various actors, including cooperatives, in transforming raw vegetables into final products. This study primarily utilizes Value Chain Theory to explore the function of cooperatives, aiming to contribute to value chain development by incorporating insights from multiple theoretical approaches - Mapanga et al. [37], Thun [39], Loni and Parand [40], Nangole et al. [41], Khodmi et al. [42], Ibrahim and Abubakar [43] and Rodríguez [44].

***Cooperative Theory****:* This theory focuses specifically on the role of cooperatives in agricultural markets. It is crucial to understand how cooperatives support smallholder farmers through collective action, shared resources, and improved bargaining power. This theory helps to explore how cooperatives contribute to the efficiency and effectiveness of the vegetable value chain by providing inputs, credit, and technical support.

***Social Network Theory****:* This theory sheds light on the relationships and social structures within the value chain. It is relevant for analyzing how cooperatives, traders, and other stakeholders interact and collaborate. Understanding these social networks helps to identify how information and resources flow between actors, and how these networks influence value addition and market access.

***Game Theory****:* This theory is used to analyze decision-making processes among actors in the value chain, especially in conflict or cooperation scenarios. It helps in understanding strategic interactions between cooperatives, farmers, and other market participants, providing insights into how decisions are made regarding pricing, investment, and market entry.

***Stakeholder Theory****:* This theory emphasizes the importance of various stakeholders in the value chain, including cooperatives, farmers, traders, and consumers. It is relevant for understanding how different stakeholder interests and power dynamics affect value chain operations and outcomes. This theory helps in assessing how cooperatives balance the interests of various stakeholders to enhance value chain performance.

***Organizational Change Theory****:* This theory examines how organizations, including cooperatives, adapt and change in response to internal and external pressures. It is important for understanding how cooperatives evolve in the context of changing market conditions, technological advancements, and policy interventions. This theory helps to analyze how organizational changes within cooperatives impact their role in the value chain.

**Fig. 1:**
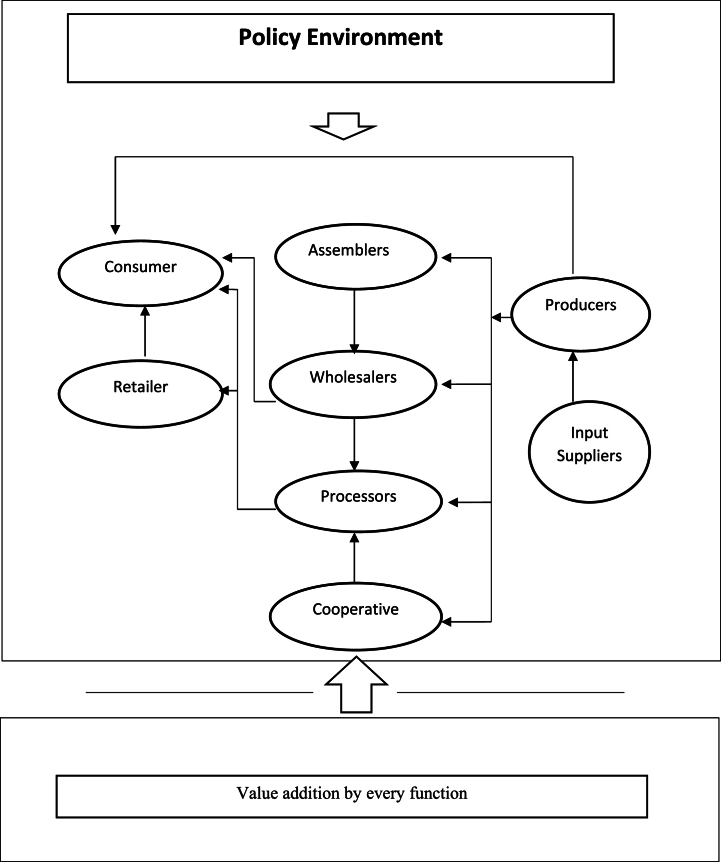
Fig. 1: Conceptual framework illustrating actors and processes in Nepal's vegetable value chain.

Source: Author's creation

Several studies have recommended vegetable value chain analysis techniques such as the value chain framework on network structure [45], the general framework for value chain actors and support system [46], the supply chain framework of the value chain model [47], the framework of the agricultural value chain and associated business development services [48]. Among them, Usman [49] assigned that the vegetable value chain can be analyzed by a general framework for value chain actors and support systems. The framework includes these dimensions which are the technical structure, the actors in a chain, the territorial, the input-output and the governance structure. In the previous studies, we see value chain analysis focuses on the actors who are directly involved in moving the physical product from the input suppliers to the end consumer. Such relations can be discussed within several actors and then the performance (as discussed in Fig. 1).

The vegetable value chain includes various actors—input suppliers, farmers, traders, and consumers—each adding value at different stages. Input suppliers provide essential agricultural inputs, while farmers handle input preparation, post-harvest handling, and selling. Traders, including collectors and assemblers, gather vegetables from farmers and resell them to wholesalers and retailers. Brokers and middlemen connect farmers to markets and other stakeholders, especially when market access is limited. Wholesalers distribute vegetables to exporters, retailers, and consumers, and retailers serve as the final link to consumers. Supporting actors offer training, information, financial, and research services. Governance of the value chain, led by key actors like wholesalers and traders, regulates the flow of goods and prices. Access to services, technology, and finance is crucial for the success of all value chain participants. Understanding these roles and interactions enhances the efficiency, profitability, and income of smallholder farmers.

### 2.2 Study area, population and sampling

**Fig. 2:**
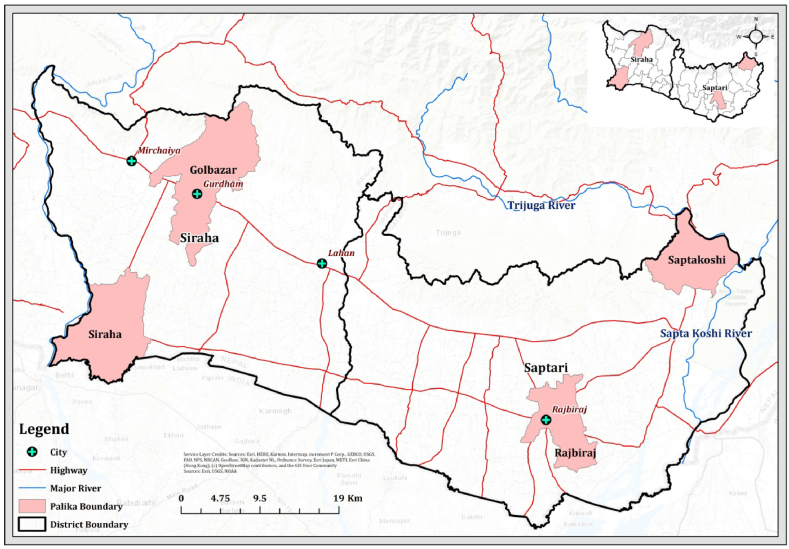
Study area.

Source: GIS

The study took place in Nepal's Saptari and Siraha districts, which were randomly selected among the high agricultural potential lands of Terai and a significant number of farmers involved in commercial vegetable production. Saptari and Siraha districts with areas of 1363 km^2^ (526 sq mi) and 1188 km^2^ (459 sq mi) have a population of 639,284 and 637,328 respectively (Fig. 2). Saptari has vegetable farming occupying 9500 ha of land primarily cultivated by smallholder farmers while in Siraha such dedicated vegetable farmland is 6165 ha. In the case of the study conducted in Siraha and Saptari districts, three municipalities - Golbazar, Saptakoshi, and Rajbiraj - were purposely selected. Specifically, wards 4 and 5 of Golbazar municipality, and wards 13 and 3 of Rajbiraj and Saptakoshi municipalities were purposely chosen. The total number of households in Wards 4 and 5 was 823 and 509 respectively, while in Wards 13 and 3 it was 806 and 236 respectively [15]. Pre-focus group discussions indicated that approximately 85 %–90 % of farmers in Golbazar municipality's wards 4 and 5, and 80 %–85 % in Rajbiraj and Saptakoshi municipality's wards 13 and 3, were engaged in vegetable farming. This study considered 85 % of the total households in the selected wards as vegetable farmers. These districts are also suitable for study areas in terms of factors like agricultural intensity, vulnerability, accessibility, and the presence of advanced agricultural technologies.

### 2.3 Sampling, data collection and analysis

Purposive sampling was employed due to the lack of reliable records, and the sample size was determined by selecting specific municipalities and wards known for their high involvement in vegetable farming, ensuring representation of the homogenous population engaged in this activity. We collected the data from Jan–May 2023.

Following Daniel [50] the Finite Population Correction Factor (FPC) was used to adjust the variance estimate when sampling without replacement with the formula:

(1) n = N∗X / (X + N – 1)

where, X = Z_α/2_^2^∗p∗(1-p)/ɛ^2^, Z_α/2_ is the critical value of the Normal distribution at α/2 (e.g. for a confidence level of 95 %, α is 0.05 and the critical value is 1.96), ɛ is the margin of error, p is the sample proportion, and N is the population size. Applying the FPC, the calculated sample size for the study was 182 households with a 95 % confidence interval and a 5 % margin of error. However, due to the COVID-19 pandemic outbreak in the study area, only A total of 164 vegetable farmers were surveyed (Table 3). Since all of them are vegetable farmers with similar socioeconomic characteristics, we believe that our sample is true representative of the larger vegetable farming population.

**Table 3:** Population and sample size for the survey municipality.

District	Municipality	Ward No.	Households	Household in Farming^a^	Sample Size
Saptari	Saptakoshi	3	236	202	361 (7.82 %)
	Rajbiraj	13	806	645	45 (6.97 %)
Siraha	Golbazar	4	823	700	50 (6.07 %)
		5	509	458	33 (6.48 %)

a (80–85) % in Saptari & (85–90)% in Siraha.

This study employs a mixed methods approach to provide a comprehensive analysis of the vegetable value chain in Nepal. The quantitative component involves statistical analysis of survey data collected from vegetable farmers, traders, and cooperative members to assess key variables influencing value addition. This includes the use of descriptive and inferential statistics to identify trends, relationships, and impacts within the value chain. Concurrently, the qualitative component involves in-depth interviews and focus group discussions with key stakeholders to gain nuanced insights into their experiences, practices, and challenges. This approach allows for a richer understanding of both the numerical patterns and the contextual factors affecting the vegetable value chain. By integrating these methods, the study aims to offer a holistic perspective on the role of cooperatives and other actors in enhancing value addition and addressing sectoral challenges.

This study involved 183 participants, including 164 farmers, 10 traders, and 9 individuals from cooperatives. Farmers' data were collected through surveys with the help of KOBO Toolbox, while traders and cooperatives were interviewed with the help of a semi-structured questionnaire using the snowball sampling method in a paper-pencil format. This was necessary because traders and cooperatives were not widely known, even among the general public. The survey data provided insights into farmers' perspectives, practices, and experiences in vegetable farming, while the interviews allowed researchers to gather information from traders and cooperatives. All validity concerns were addressed at the appropriate stages to ensure the data met the necessary validity and reliability standards. Microsoft Excel and STATA (Version 16) were used to perform the inferential analysis.

### 2.4 Logistic regression

The binary logistic regression model was chosen for this study due to its suitability for analyzing categorical dependent variables, particularly binary outcomes. In this context, the dependent variable is whether a farmer participates in value addition or not, which is a dichotomous variable (1 if the farmer participates, 0 otherwise). In this study, *Y* is the available adaptation option to the farmer, which is a random variable, and X represents socioeconomic, institutional, social and other factors [51]. The inferential statistical analysis used in this study is a binary logit model for such a dichotomous outcome. The effect of X on the response probabilities, *P(y = j/x)*, can be estimated using a binary logit model, which is expressed as:

$$P\left(Y_{i}/X\right)=F\left(Z_{j}\right)=\frac{e^{z}i}{1+e^{z}i}=\frac{1}{1+e^{‐z}i}$$

$$P\left(Y_{i}=J/X_{i}\right)=F\left(Z_{j}\right)=\frac{e^{z}i}{1+e^{z}i}=\frac{1}{1+e^{‐z}i}$$

(1) $$Z_{i}=\beta_{0}+\beta_{1}X_{1i}+\ldots +\beta_{n}X_{\text{ni}}+\mu_{i}+$$

The logit regression equation that is used to ascertain variables influencing determinants of value addition on vegetable value chain is:

(2) $$Y=\beta_{0}+\beta_{1}X_{1}+\beta_{2}X_{2}+\beta_{3}X_{3}+\beta_{4}X_{4}+\beta_{5}X_{5}+\beta_{6}X_{6}+\ldots +\beta_{n}X_{n}+\mu_{0}$$

where P = probability of the outcomes, β_0_ = intercept term, β_1_ … β_n_ = coefficient, X_1_ … X_n_ = independent variables.

The study used purposive sampling to focus on those wards with 85 % household involvement in vegetable farming, while snowball sampling identified key traders and cooperative members due to a lack of records. It assumes the sample represents the broader farming population, with independent responses and a linear relationship between variables, despite a reduced sample size of 164 farmers due to COVID-19. The model is presumed to include all relevant variables influencing value addition.

### 2.5 Variables and their definitions

Table 4 shows the details of the variables used in the study.

**Table 4:** Variables and their expected signs.

Variables	Description	Value	Sign
***Dependent Variable***			
Factors affecting value addition	Represents whether the farmer participates in value addition or not.	1 if the farmers participate in value addition, 0 otherwise.	±
***Independent Variables***			
Membership to cooperative	Farmers who are members of cooperative	1 if the household is member of cooperative, 0 otherwise	+
Access to market information	Vegetable producer gets market information	1 if farmer accesses to market information, 0 otherwise	+
Access to extension service	Household had access to extension services	1 if a household had access to extension services, 0 otherwise	+
Size of farmland	Total area of farmland a farmer owned, rented in and/or shared	Continuous (Katha)	+
Livestock	Farmers who have many livestock.	Continuous (Livestock unit (LU).	±
Farming experience	Household engaged in farming activities	Continuous (years)	±
Education level	Formal schooling of the household	1 if formal education, 0 if illiterate	+
Family size	Number of members in a household	Continuous (number)	+
Age	Age of respondent	In years	+
Distance to nearest market	The closer a household to the nearest urban center	Continuous (Km)	+
Inputs	Source of input and access to input would enhance the production capacity of the farmer	1 if yes, 0 otherwise	+
Storage	Household with storage facility	Continuous (Days)	+
Training	Household that had training and related activities	1 if yes, 0 Otherwise	+
Family type	The types of family	1 nuclear, 2 joint, 3 extended	±
Income from non/off farm activities	Household obtained income from off/nonfarm activities	1 if yes, 0 otherwise	+
Access to credit	Access to credit enhances the financial capacity of the farmer	1 if the household takes loan, 0 otherwise	+
Ownership of market transport facilities	Specifically, vehicles, carts and transport animals	1if household owned transportation facilities, 0 otherwise	+

Table 4 presents the variables used in the study to examine factors influencing value addition in the vegetable value chain. The dependent variable, "Factors affecting value addition," indicates whether farmers participate in value addition (coded as 1) or not (coded as 0). The independent variables include membership in cooperatives, access to market information, extension services, farm size, livestock ownership, farming experience, education level, family size, age, distance to the nearest market, access to inputs, storage capacity, training participation, family type, income from non/off-farm activities, access to credit, and ownership of market transport facilities. Each variable is described with its respective value type and expected positive or negative sign, reflecting its impact on value addition.

## 3 Results and discussions

This section provides a detailed analysis of the findings of the study. The descriptive analysis summarizes the socio-demographic characteristics, current status of farming, and challenges faced along the value chain, while the inferential analysis examines the relationship between variables. Moreover, the application of binary logistic regression provides insights into the factors affecting the vegetable value chain, which will aid in developing effective policies and strategies to improve the vegetable value chain in the study area.

### 3.1 Descriptive analysis

#### 3.1.1 Socio-demographic characteristics

In this study, 164 respondents from three municipalities were interviewed, with 83, 45, and 36 respondents from Golbazar, Saptakoshi, and Rajbiraj, respectively. The socio-demographic characteristics of farmers are shown in Table 5.

**Table 5:** Socio-demographic characteristics of farmers.

Title	Specifics	Frequency	Percentage
Sex	Male	116	71
	Female	48	29
Marital Status	Married	140	85
	Single	19	12
	Others	6	3
Address	Golbazar	83	51
	Saptakoshi	36	22
	Rajbiraj	45	27
Education	level +2	60	37
	Bachelor	50	30
	SLC/SEE	26	16
	Lower secondary	12	7
	None	6	4
	Primary	6	4
	Master and above	4	2
Type of family	Nuclear	80	49
	Joint	58	35
	Extended	28	16
Age	Below 30	18	11
	30–40	56	34
	41–50	43	26
	Above 50	47	29
Family members	Below 5	19	12
	5 to 7	92	56
	8 to 11	40	24
	12 & above	13	8
Farming Experience	Below 10	18	11
	10 to 15	98	60
	16 to 20	35	21
	Above 20	13	8

The study reveals valuable insights into the characteristics and practices of vegetable farmers and traders in Golbazar, Saptakoshi, and Rajbiraj municipalities of Nepal. It shows a shift towards education and away from traditional farming, with a high literacy rate and an increase in nuclear families. Farmers generate income from various sources, including crop sales, off-farm activities, and livestock sales. Household heads, particularly fathers and husbands, make most of the decisions in vegetable production, while experienced farmers play a crucial role in the market supply. Gender disparity is observed in vegetable trading, with all traders being male, emphasizing the need for greater female participation. The study also highlights the growth of cooperatives, indicating increased interest in collective action for improved market access and bargaining power. A study by Fernqvist and Göransson [1] also demonstrates similar results regarding the level of education, household heads, and gender disparity in creating the vegetable value addition.

#### 3.1.2 Current status of farming

The study surveyed vegetable farmers to provide an overview of farming in a specific area. It found that most farmers were engaged in vegetable production, with onion, potato, and tomato being the most commonly grown crops. Tomato production was lower due to disease and high temperatures. The average land size for vegetable farming is small, and farmers rely on family and relatives for cultivation knowledge. Education and training were seen as ways to increase productivity. Accessing inputs was a major challenge due to supply shortages, high costs, and remote locations. These problems were attributed to governance issues and coordination among value chain actors.

The provision of adequate services, such as credit, extension services, and market information, is crucial for socioeconomic development and individual well-being. In the study area, 85.63 % of respondents have access to credit, primarily for purchasing farm inputs. Cooperatives, microfinance, and other institutions are the main sources of credit. However, inadequate supply and collateral pose challenges. Most farmers use irrigation facilities (76.65 %) and rely on family and hired labor. Most vegetables are sold at harvest, with a small portion kept for home consumption and seed. Enhancing access to education, training, inputs, credit, and irrigation can improve productivity, marketability, and income for smallholder farmers [52].

#### 3.1.3 Challenges in the production and marketing of vegetables

The results indicate that the major constraints impeding the development of the vegetable value chain can be categorized into two stages: the farm level and the marketing/traders stage.

Table 6 outlines the production and marketing challenges within the vegetable value chain, as identified by a survey. Production issues include insect infestations, diseases, lack of credit and irrigation, inadequate inputs, limited technical support, and insufficient government aid. Marketing difficulties cover low prices at the farm gate, price volatility, payment delays, poor processing and storage, transportation problems, scarce market information, crop rejection, middlemen's role, and low production levels. These findings are in line with Rahman et al. [53], highlighting similar post-harvest, pest control, storage, and input challenges, and Baral et al. [24], noting input access issues and loss due to poor post-harvest handling and pest/disease management training. Additionally, Sharma [19] and Mazhar et al. [33] corroborate the marketing constraints of low prices, high costs, weak links, limited bargaining power, market instability, and storage issues, affecting quality and leading to buyer concerns over transportation, cost, and storage. Cooperatives also face transparency, management, structure, and market opportunity challenges. The survey indicates these challenges are moderate to severe, necessitating a collective approach from all value chain participants for resolution.

**Table 6:** Production and marketing problem.

Problems	Severe (%)	Moderate (%)	Slight (%)	No (%)
**Production**				
Infestation of Insect and Diseases	14.6			
Lack of Credit Facility	5.5	76.4	7.3	1.8
Lack of Irrigation Facilities	6.7	20.6	67.3	6.7
Lack of Quality Input	7.3	70.9	19.4	3.1
Supply of Inputs in Appropriate Time	10.3	65.5	25.5	1.8
High Cost of Inputs	4.2	72.1	12.7	4.9
Unavailable Farm Labor	6.7	54.6	40.6	0.6
High Labor Wage	4.9	40.00	43.6	9.70
Lack of Technical Knowledge Support	4.9	27.9	63.0	4.2
Inadequate Government Support	18.8	67.9	23.0	4.2
**Marketing**				
Low Farm Gate Price	7.3	80.6	10.9	1.2
High Price Fluctuation	2.4	37	58.2	2.4
No Timely Payment	3.0	77	13.3	6.7
Insufficient Processing Facilities	6.1	64.9	27.3	1.8
Insufficient Storage Facilities	6.1	70.3	21.8	1.8
Transportation Problem and Inaccessible Market	2.4	33.3	57.6	6.7
Insufficient Market Information (About Price and Quality)	1.2	47.9	49.1	1.8
Rejection of Crop by Traders Reasoning Low Quality	2.4	37	58.8	1.8
Presence of Middleman	1.8	30.9	63.6	3.6
Low Production	4.85	41.21	50.30	3.64

### 3.2 Managerial implications

The findings of this study offer several important managerial implications for stakeholders involved in the vegetable value chain in rural Nepal. Firstly, addressing the gender disparity in vegetable trading through targeted training programs and incentives can enhance female participation, thereby diversifying market dynamics and potentially increasing profitability. Secondly, improving cooperative management and transparency is crucial despite the identified negative impact on value addition, suggesting a need for streamlined governance structures and enhanced support services to optimize cooperative effectiveness. Thirdly, facilitating better access to inputs, credit, and market information can mitigate production and marketing challenges, fostering a more resilient and competitive value chain. Lastly, investing in education and technical training for farmers can significantly boost productivity and marketability, aligning with long-term sustainable development goals in agricultural practices.

#### 3.2.1 Theoretical implications

Theoretical implications drawn from this study contribute to the broader field of agricultural economics and value chain analysis. The findings underscore the significance of cooperative governance in rural development contexts, highlighting both its potential benefits in collective action and its challenges in optimizing value addition. Moreover, the study reinforces theories on human capital development by illustrating how education and training influence farmers' ability to adopt modern agricultural practices and integrate into market economies. Additionally, the emphasis on access to market information and credit resonates with theories of information economics, demonstrating how asymmetric information can impact market efficiency and farmer livelihoods. Overall, these theoretical insights deepen our understanding of the factors shaping agricultural value chains in developing regions, offering pathways for future research and policy interventions aimed at sustainable agricultural development.

### 3.3 Inferential analysis

#### 3.3.1 Diagnostic checking

*Pre-estimation test:* Pre-estimation tests identify issues to adjust and improve accuracy. Details of the model fit are shown in different headings. The _hat value is 0.000 and _hatsq value is 0.23. So, we can conclude that the chosen predictors χ were meaningful. The result obtained for the model is Prob > χ^2^ = 1.00 which is greater than 5 % so we can say that there is a goodness of fit in our models. The count R^2^ for the model is 0.92 which is higher than 0.7, so it's excellent.

*Post-estimation test:* On the other hand, post-estimation tests in econometrics evaluate the goodness of fit and diagnose potential problems after model estimation. Multicollinearity and heteroscedasticity tests are conducted in this case, as it is not time series data with no autocorrelation. The variance inflating factor as per the calculation for the model (Mean VIF) is 2.15. As we know if VIF is greater than 10, there exists multicollinearity. So, we can say that there is no multicollinearity in our data set. Looking towards the hettest, the result that appeared for the model is prob > χ^2^ = 0.0000. The assumptions show that there is a presence of heteroscedasticity if the value is less than 0.05. So, there is the presence of heteroscedasticity in the case of the model. In a nutshell, this data shows there is no problem besides the problem of heteroscedasticity. Hence, while performing final estimation robust standard error test has been performed.

#### 3.3.2 Logistic regression results

The result shows that the value pf Wald χ^2^ (31) is 55.60 which explains that our model is fit and we can go ahead. Moreover, the pseudo-R^2^ of 0.6115 suggests strong explanatory power, aligning with Cohen's [54] guidelines, which consider R^2^ values above 0.26 as indicative of a high effect size.^1^ It indicates that the independent variables, undertaken for the study are explained as dependent variables by 61.15 %. It is very good in terms of logistic regression. According to Cohen [54], an R^2^ value of 0.12 or below indicates low, between 0.13 and 0.25 values indicate medium, and 0.26 or above, and above indicates high effect size. Similarly, the log pseudolikelihood value is −24.38. The coefficient value of a constant is −10.52. Notably, this model's explanatory power surpasses that of similar studies in the field. For instance, Mwangi et al. [55], in their study on vegetable value chains in Tanzania, reported a pseudo R^2^ of 0.31. Similarly, research conducted by Ochieng et al. [56] on smallholder vegetable farmers in Kenya found a pseudo-R2 of 0.28. The comparatively higher explanatory power of the current model suggests that it captures a more comprehensive set of factors influencing value addition in the Nepalese context, potentially offering more robust insights into the dynamics of vegetable value chains in the region.

The binary logit model presented in Table 7 shows fourteen significant variables that affect value addition in the vegetable industry. The marginal effect analysis reveals that cooperative membership is associated with a reduction in the likelihood of value addition by 18.5 percentage points. In a similar vein, Amaya et al. [34] also found that variables like membership to cooperatives, total land, and input from cooperatives, among others have a negative marginal effect on value addition. Similarly, each additional year of an individual's age is correlated with a 2.07 percentage point increase in the probability of value addition. Furthermore, possessing off-farm income is linked to a 25.7 percentage point enhancement in the likelihood of value addition. Conversely, each additional unit of land owned relates to a marginal decrease of 0.557 percentage points in the probability of value addition, whereas allocating an additional unit of land for vegetable cultivation is associated with a 1.82 percentage point increase in the likelihood of value addition. Utilizing inputs sourced independently is correlated with a substantial 31.8 percentage point increase in the probability of value addition. Accessing information via the internet is associated with a notable 35 percentage point increase in the likelihood of value addition.

**Table 7:** Results of logistic regression.

Variables	Dependent Variable: Factors affecting value addition		
	Logit Model	Odds Ratio	Marginal Effects
Membership to cooperatives	−3.962∗∗ (1.723)	0.0190∗∗ (0.0328)	−0.185∗∗ (0.0806)
Age	0.442∗∗∗ (0.145)	1.555∗∗∗ (0.226)	0.0207∗∗∗ (0.00704)
Family member	0.463 (0.290)	1.589 (0.461)	0.0216∗ (0.0131)
Level of education	0.587	1.798	0.0274
	(0.461)	(0.828)	(0.0226)
Family types	−1.013	0.363	−0.0473
	(0.789)	(0.287)	(0.0391)
Farming experience	−0.199	0.820	−0.00928
	(0.122)	(0.0998)	(0.00566)
Off farm income	5.502∗∗	245.2∗∗	0.257∗
	(2.752)	(674.9)	(0.133)
Total land	−0.119∗∗	0.888∗∗	−0.00557∗∗
	(0.0483)	(0.0429)	(0.00235)
Land allocated for vegetable	0.390∗∗	1.477∗∗	0.0182∗∗
	(0.164)	(0.242)	(0.00764)
Input from own source	6.799∗∗	896.6∗∗	0.318∗∗
	(3.193)	(2863)	(0.146)
Input from agro-vet services	2.502	12.21	0.117
	(2.416)	(29.51)	(0.110)
Input from cooperatives	−3.065∗	0.0467∗	−0.143∗
	(1.754)	(0.0818)	(0.0777)
Input locally available	1.378	3.966	0.0644
	(2.651)	(10.51)	(0.121)
Input from GOs/NGOs	−3.959	0.0191	−0.185∗
	(2.439)	(0.0465)	(0.111)
Input quantity at the right time	−4.888∗∗	0.00753∗∗	−0.229∗∗
	(2.189)	(0.0165)	(0.102)
Access to credit	−3.361∗	0.0347∗	−0.157∗
	(1.723)	(0.0598)	(0.0829)
Problem in getting credit	5.100∗∗∗	164.0∗∗∗	0.238∗∗∗
	(1.267)	(207.8)	(0.0546)
Transportation from vehicle	−0.881	0.414	−0.0412
	(1.529)	(0.633)	(0.0707)
Ownership of transportation	−1.374	0.253	−0.0642
	(2.440)	(0.618)	(0.115)
Information from radio	−3.329∗	0.0358∗	−0.156∗∗
	(1.705)	(0.0611)	(0.0783)
Information from Newspaper	2.064∗	7.879∗	0.0965∗∗
	(1.176)	(9.262)	(0.0474)
Information from TV	−2.440	0.0872	−0.114
	(1.613)	(0.141)	(0.0713)
Information from cooperatives	0.992	2.696	0.0464
	(1.937)	(5.221)	(0.0888)
Information from vegetable traders	2.867∗	17.59∗	0.134∗
	(1.522)	(26.77)	(0.0703)
Information from internet	7.483∗∗∗	1778∗∗∗	0.350∗∗∗
	(2.722)	(4839)	(0.122)
Information for mobile SMS	−1.996	0.136	−0.0933
	(2.169)	(0.295)	(0.108)
Nearest market	−1.126	0.324	−0.0526
	(0.865)	(0.281)	(0.0397)
Extension service	−0.0610	0.941	−0.00285
	(1.198)	(1.127)	(0.0559)
Ownership of livestock	0.993	2.698	0.0464
	(1.270)	(3.427)	(0.0613)
Training	−0.437	0.646	−0.0204
	(1.004)	(0.648)	(0.0462)
Days of Storage	0.336∗∗∗	1.399∗∗∗	0.0157∗∗
	(0.125)	(0.175)	(0.00645)
Constant	−10.52∗∗∗	2.69e-05∗∗∗	
	(3.978)	(0.000107)	
Observations	164	164	164

Robust standard errors in parentheses, ∗∗∗p < 0.01, ∗∗p < 0.05, ∗p < 0.1.

Similarly, Each additional day that produce is stored is linked to a 1.57 percentage point increase in the probability of value addition. Receiving information from vegetable traders and newspapers is associated with increases of 13.4 and 9.65 percentage points, respectively, in the likelihood of value addition. In contrast, receiving inputs from cooperatives is connected with a 14.3 percentage point decrease in the probability of value addition. Ensuring the right quantity of input at the correct time is associated with a 22.9 percentage point decrease in the likelihood of value addition. Lastly, access to credit is correlated with a 15.7 percentage point reduction in the probability of value addition. Access to information, such as from newspapers and traders, has a positive impact on production and increases the odds of value addition by 7.87 and 17.59 times respectively [57]. The odds ratio for information from the internet, which provides valuable farming information, is 1.7 times. Additionally, increasing the duration of storage by a day enhances the odds of value addition by 1.399 times. This shows that these significant variables do affect value addition in vegetables because these results are not due to random variation but have a strong chance of influencing factors affecting value addition. However, a study conducted in Bangladesh Rahman et al. [58], found such variables have only a partial effect on the value chain process.

The study reveals intriguing contrasts with existing literature. Cooperative membership shows a surprisingly negative effect on value addition in Nepal, unlike positive impacts found in Ethiopia [59] and China [60]. Age's positive effect aligns with findings from Kenya [61] but differs from Zimbabwe [62]. The strong positive impact of off-farm income on value addition echoes studies in Kenya [63] and Ethiopia [64], suggesting it may provide capital for value-adding investments in Nepal. These findings highlight the importance of context-specific research in understanding agricultural value chains.

The study reveals nuanced findings regarding land allocation, information sources, and credit access in Nepal's vegetable value chains. Land allocated specifically to vegetables shows a positive effect on value addition, contrasting with the negative effect of total land ownership. This aligns with Olwande et al.'s findings in Kenya [65], where land allocation was more influential than total land owned for commercialization. Information sources play a crucial role, with internet access, vegetable traders, and newspapers significantly increasing value addition. These results echo studies by Muthini et al. [66] in Kenya and Ochieng et al. [67] in Tanzania, which found positive impacts of market information and mobile phone use on commercialization and market participation. However, the negative effect of radio information in Nepal is unexpected and warrants further investigation. Surprisingly, credit access shows a negative effect on value addition, contradicting findings from Kenya [68] and Ghana [69] where credit access increased market orientation and participation. Again, This unexpected result may indicate issues with local credit systems or terms in Nepal that discourage value-addition investments, highlighting the need for context-specific research and targeted policy interventions in agricultural value chains.

The findings not only offer insights into the effectiveness of cooperative-backed vegetable value chains in Nepal but also contribute to the theoretical understanding of value chain integration in developing economies. Specifically, the results challenge the traditional view of cooperative structures as sole facilitators of economic efficiency by demonstrating their role in enhancing social capital and local governance. This extends the application of social capital theory in the context of agricultural cooperatives, suggesting that future research should explore the multidimensional impacts of cooperatives beyond economic outcomes.

#### 3.3.3 Value chain analysis of vegetables in the study area

**Fig. 3:**
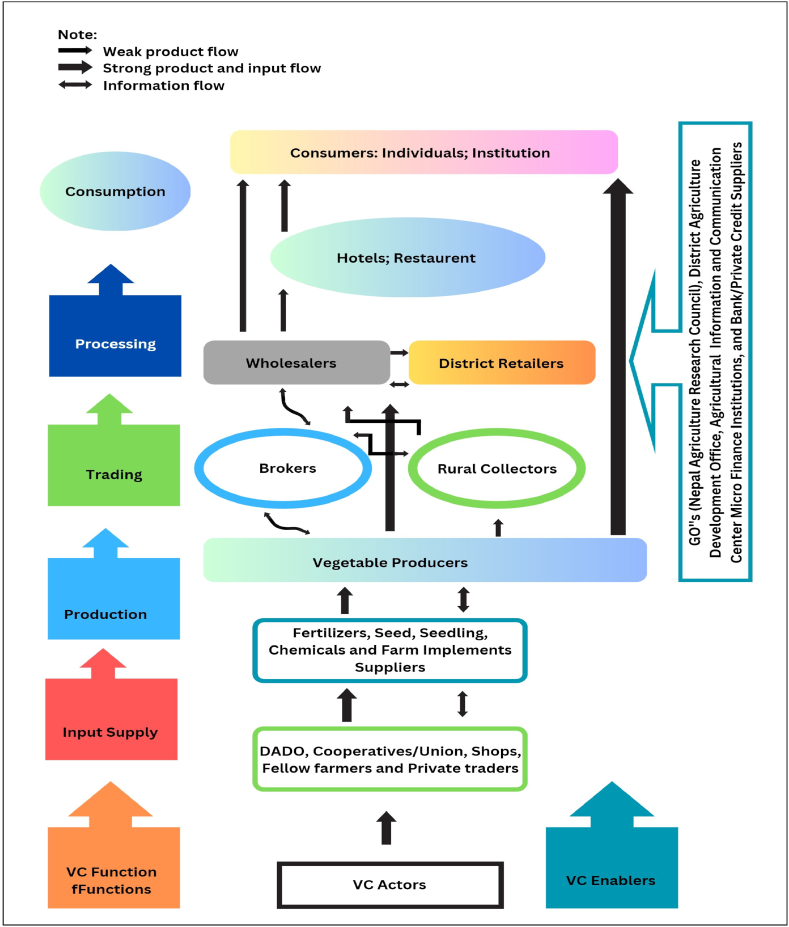
Value Chain map of vegetables in the study area.

Source: Authors sketch from the survey result

The qualitative analysis identifies factors influencing the organization's value chain activities by assessing primary and support activities based on value chain actors' reports. The study creates a value chain map (Fig. 3) using respondents' opinions, illustrating interconnected functions and processes in the vegetable value chain. The map emphasizes the significance of input supply, production, trade, processing, and consumption.

The vegetable value chain in Siraha and Saptari districts involves a range of stakeholders, from input suppliers to consumers, each adding value through their roles. Input suppliers, including cooperatives and NGOs, provide essential materials, with many farmers using their seeds. Producers face low prices and post-harvest losses, with most using irrigation. Collectors and brokers are key in linking farmers to markets, while wholesalers act as major hubs, purchasing and stocking vegetables. Retailers connect producers to consumers, who prioritize quality and influence the chain's dynamics. Supporting actors like the District Agricultural Development Office, Nepal Agricultural Research Council, Department of Agriculture, agricultural information centers, primary cooperatives, microfinance institutions, and NGOs offer training and credit, essential for the chain's success. Wholesalers and brokers dominate governance, with farmers having limited bargaining power. Enhancing market information and supportive policies is crucial for improving smallholders' market positions.

In the vegetable value chain of Siraha and Saptari, Nepal, various actors were identified, including input suppliers, farmers, traders, and consumers. Farmers are the primary actors responsible for input preparation, post-harvest handling, and selling, but face challenges due to low prices and losses. Cooperatives play a significant role in providing inputs and access to credit. Collectors and brokers connect farmers to markets, while wholesalers assemble and distribute vegetables. Retailers transport, grade, exhibit, and sell to consumers. Collaboration among these actors is crucial for a successful value chain [70,71].

The study highlights the importance of collaboration among actors in the vegetable value chain in Siraha and Saptari. It emphasizes the role of cooperatives in providing inputs and credit to farmers. Collectors and brokers facilitate market access, while wholesalers serve as assembly centers. Retailers bridge the gap between wholesalers and consumers [72]. The study recommends improving efficiency and addressing factors affecting the value chain. The findings offer insights for policy development and programs to support farmers and enhance the profitability and livelihoods within the vegetable value chain.

## 4 Conclusions, policy implications and future research

This study provides a comprehensive analysis of the vegetable value chain in Golbazar, Saptakoshi, and Rajbiraj municipalities of Nepal, highlighting the socio-demographic characteristics, current farming status, and challenges faced along the value chain. The findings from both descriptive and inferential analyses reveal several critical factors influencing the vegetable value chain and offer insights for enhancing productivity, marketability, and overall value addition. The descriptive analysis indicates a shift towards education and nuclear family structures among farmers, with a significant reliance on household heads for decision-making. Despite high literacy rates and the involvement of experienced farmers in market supply, the gender disparity in vegetable trading remains a notable issue. The current status of farming shows that while most farmers engage in vegetable production, they face challenges related to input access, credit availability, and irrigation. Additionally, significant constraints in production and marketing, such as pest infestations, inadequate technical support, and poor market infrastructure, hinder the development of the vegetable value chain.

The inferential analysis, particularly the application of binary logistic regression, identifies key variables affecting value addition. Cooperative membership, age, off-farm income, land allocation for vegetables, independent input sourcing, and access to information significantly influence the likelihood of value addition. The model's strong explanatory power underscores the importance of these variables in understanding the dynamics of the vegetable value chain in the study area. Based on the findings of this study, several policy implications emerge to improve the VVC. Policies should promote greater female participation in the value chain through targeted training programs, support services, and incentives, while also strengthening cooperative structures by enhancing management, transparency, and support services. Addressing challenges related to input shortages, high costs, and credit access is essential, necessitating better coordination among value chain actors, improved supply chain logistics, and tailored financial products for smallholder farmers. Enhancing farmers' education and training through agricultural programs, extension services, and technical training can significantly boost productivity and marketability. Developing robust market information systems via the Internet, mobile SMS, and traditional media is crucial for informed decision-making and value addition. Furthermore, improving infrastructure such as irrigation facilities, storage systems, and transportation networks will reduce post-harvest losses and enhance market access. Promoting diversification in crop production and encouraging innovative practices will enhance resilience and value addition, supported by research and development initiatives, incentives for new technologies, and facilitated knowledge sharing among farmers. By addressing these policy implications, stakeholders can work collectively to overcome challenges in the VVC and promote sustainable agricultural development in the study area.

While this study provides valuable insights into the vegetable value chain in the rural areas of Siraha and Saptari districts of Nepal, several limitations should be acknowledged. Firstly, the study's sample size was relatively small, comprising respondents from specific municipalities, which may limit the generalizability of the findings to broader contexts within Nepal or other regions. Moreover, the reliance on questionnaire surveys and semi-structured interviews could introduce biases in data collection, such as respondent bias or social desirability bias. Future research could address these limitations by employing larger and more diverse samples to enhance representativeness and validity. Additionally, exploring the impact of seasonal variations on the vegetable value chain and conducting longitudinal studies could provide deeper insights into the dynamics of value addition over time. Furthermore, investigating the role of technological interventions, such as digital platforms for market information dissemination or blockchain for supply chain transparency, could offer innovative solutions to improve efficiency and sustainability in vegetable value chains. These avenues for future research could contribute to a more comprehensive understanding and enhancement of agricultural value chains in similar rural contexts.

## CRediT authorship contribution statement

**Ghanashyam Khanal:** Writing – review & editing, Methodology, Formal analysis, Conceptualization. **Ratnesh Kumar Dev:** Writing – original draft, Project administration, Methodology, Formal analysis, Data curation, Conceptualization. **Tek Maraseni:** Writing – review & editing, Validation, Supervision, Methodology, Formal analysis. **Niranjan Devkota:** Writing – review & editing, Writing – original draft, Visualization, Validation, Supervision, Software, Methodology, Formal analysis, Data curation, Conceptualization. **Udaya Raj Paudel:** Writing – original draft, Supervision, Methodology, Formal analysis, Conceptualization.

## Consent to participate

As per mentioned in the questionnaire – informed consent was obtained from the participant to collect the questionnaire. And, there are no any/all images, clinical data and other data included in the manuscript which requires participants' consent in order to publish. Also, there are no participants under the age of 18. It can be verified from the dataset used in this study. We have only 18 people aged below 30 and for sure none with age below 18.

## Ethical approval

Quest International Review Committee (QIRC) approved the research on April 10, 2021. The reference number for ethical approval is 115.

## Data availability statement

All data collected is used for analysis purposes. The dataset will be provided on request.

## Funding

This research did not receive any specific grant from funding agencies in the public, commercial, or not-for-profit sectors.

## Declaration of competing interest

The authors declare that they have no known competing financial interests or personal relationships that could have appeared to influence the work reported in this paper.

## Footnotes

1 The pseudo-R^2^ value provides a measure of how well the model explains the variability in the data for models like logistic regression, indicating the proportion of variation in the dependent variable that is captured by the independent variables.
